# Revitalizing Trimethoprim/Sulfamethoxazole via Nanotechnology for Improved Pharmacokinetics and Antibacterial Efficacy

**DOI:** 10.3390/antibiotics15030283

**Published:** 2026-03-10

**Authors:** Yaxin Zhou, Jing Xu, Guonian Dai, Bing Li, Weiwei Wang, Bintao Zhai, Shulin Chen, Jiyu Zhang

**Affiliations:** 1 Lanzhou Institute of Husbandry and Pharmaceutical Sciences, Chinese Academy of Agricultural Sciences, Lanzhou 730050, China; zhouyaxin@caas.cn (Y.Z.); 82101221334@caas.cn (J.X.); dai940910@163.com (G.D.); pharm2005bl@126.com (B.L.); weiweiwang1990@163.com (W.W.); zhaibintao@163.com (B.Z.); 2 College of Veterinary Medicine, Northwest Agriculture & Forestry University, Yangling 712100, China; 3 Key Laboratory of New Animal Drug Project of Gansu Province, Lanzhou 730050, China; 4 Key Laboratory of Veterinary Pharmaceutical Development, Ministry of Agriculture, Lanzhou 730050, China

**Keywords:** trimethoprim/sulfamethoxazole, polymeric nanoparticles, pharmacokinetics, bioavailability enhancement, antibacterial efficacy

## Abstract

**Objective**: The therapeutic efficacy of the classic antibiotic combination trimethoprim/sulfamethoxazole (TMP/SMZ) is often limited by the significant pharmacokinetic mismatch. In this study, a polyethylene glycol-polylactic-co-glycolic acid (PEG-PLGA) nanodelivery system was employed to improve the pharmacokinetic matching of TMP and SMZ. The investigation also evaluated the enhanced in vivo antibacterial efficacy of this formulation. **Methods**: Ultra-High Performance Liquid Chromatography–Tandem Mass Spectrometry (UPLC-MS/MS) was employed to systematically characterize the absorption, distribution, and excretion profiles of PEG-PLGA-loaded TMP nanoparticles (NPs) in rats. In vitro antibacterial activity was assessed against *Escherichia coli* (*E. coli*) and *Staphylococcus aureus* (*S. aureus*). In vivo efficacy and biosafety of the TMP NPs/SMZ regimen were evaluated using a murine *E. coli* infection model via survival monitoring, biochemical assays, and histopathology. **Results**: Pharmacokinetic analysis revealed that TMP NPs achieved a relative bioavailability of 193.05% and extended the elimination half-life by 3.37-fold compared to free TMP. Tissue distribution showed significantly increased drug accumulation in the liver, spleen, and kidneys, with renal clearance as the primary excretion pathway (73.89%). In vitro, the nano-formulation reduced the minimum inhibitory concentration (MIC) by 2-4-fold and shortened the bactericidal duration from 12 to 8 h. In vivo, the TMP NPs/SMZ combination significantly improved survival rates, accelerated recovery, and alleviated infection-induced organ damage without systemic toxicity. **Conclusions**: This nanotechnology-based strategy effectively aligns the pharmacokinetics of TMP and SMZ, prolongs their synergistic window, and enhances biosafety, offering a viable approach to revitalize classic antibiotic combinations.

## 1. Introduction

Trimethoprim (TMP), a synthetic broad-spectrum diaminopyrimidine, functions by selectively inhibiting dihydrofolate reductase. This blockade disrupts the conversion of dihydrofolate to tetrahydrofolate, thereby arresting bacterial DNA, RNA, and protein synthesis [1]. Since its introduction in the 1960s, TMP has remained a mainstay in both human and veterinary medicine. Its classification as a Class A drug in China’s National Basic Medical Insurance Drug List underscores its enduring therapeutic relevance. Clinically, TMP is a first-line treatment for urinary and respiratory infections and *Pneumocystis jirovecii* pneumonia in humans [2,3], and it also controls respiratory and enteric outbreaks in livestock [4,5]. Rarely employed as monotherapy, TMP is typically co-administered with sulfonamides at a 1:5 ratio. This strategy targets sequential steps in folate metabolism, generating a synergistic bactericidal effect that transcends mere bacteriostasis and suppresses resistance [6]. However, the clinical potential of TMP is compromised by its physicochemical profile. Classified as a Biopharmaceutics Classification System Class II compound, TMP exhibits high permeability but poor water solubility (~0.4 mg/mL at 20 °C) [7]. Consequently, dissolution becomes the rate-limiting step in the gastrointestinal tract, resulting in low oral bioavailability and significant inter-subject variability.

A critical barrier to optimal therapy is the pharmacokinetic mismatch between TMP and sulfonamides. In vivo studies reveal a significant disparity in elimination rates across species; for example, in pigs, the half-life of sulfamonomethoxine (~14 h) is far longer than that of TMP (~3 h). This asynchronous elimination prevents the maintenance of a synergistic drug ratio in the bloodstream. Consequently, TMP concentrations frequently fall below the minimum inhibitory concentration (MIC), while sulfonamide levels remain therapeutic [8]. To compensate, clinical protocols often require higher dosages or shorter dosing intervals. Such regimens, however, inflate treatment costs and heighten the risk of drug accumulation-induced toxicities, specifically nephrotoxicity and myelosuppression, while aggravating environmental antibiotic contamination [9]. Therefore, developing a delivery system that simultaneously enhances TMP solubility and synchronizes its pharmacokinetic profile with sulfonamides is essential to maximize the therapeutic index and minimize adverse effects.

To address TMP’s poor water solubility, various formulation and crystal engineering strategies have been explored. For instance, lyophilized inclusion complexes of TMP with hydroxypropyl-β-cyclodextrin (HP-β-CD) effectively optimize solid-state properties, enhancing both dissolution rate and thermal stability [10]. Similarly, pharmaceutical cocrystals of TMP with organic acids (e.g., oxalic acid) modify the crystal lattice to improve dissolution performance [11]. These approaches are supported by thermodynamic studies of host-guest interactions, which theoretically validate the utility of supramolecular carriers for poorly soluble drugs [12]. However, translating in vitro solubility into in vivo bioavailability remains a hurdle. Our previous work addressed this by developing polyethylene glycol-polylactic-co-glycolic acid (PEG-PLGA)-loaded TMP nanoparticles (NPs), which utilized an amphiphilic core–shell structure to enhance solubility and extend circulation time, resulting in a 2.82-fold increase in oral bioavailability [13]. Nevertheless, pharmacokinetic improvement of TMP as a single agent does not guarantee the restoration of synergy when delivered with sulfonamides. The disparity in elimination kinetics, specifically the mismatch between the short half-life of TMP and the prolonged clearance of sulfonamides, can disrupt the synergistic ratio essential for efficacy [14]. Even with improved absorption, the rapid elimination of TMP may lead to a loss of synergy if its retention does not match that of sulfonamides. Current literature predominantly characterizes solubility and plasma concentrations, leaving the tissue distribution and excretion kinetics of solubilized TMP underexplored. Elucidating the accumulation of nanoformulated TMP in target tissues and its clearance rates is critical for verifying spatiotemporal synchronization with sulfonamides [15,16]. Such data are indispensable for determining whether the nanocarrier strategy can overcome TMP’s inherent pharmacokinetic limitations to sustain therapeutic synergy.

Building on our previous development of TMP NPs, this study investigates the in vivo and in vitro synergy of this delivery system combined with sulfamethoxazole (SMZ) [13]. Our primary objective was to determine if the nanocarrier enhances the standard TMP/SMZ regimen by synchronizing the pharmacokinetics and pharmacodynamics of the two drugs. We characterized the absorption, distribution, and excretion profiles of the TMP NPs/SMZ combination in rats. Specifically, we compared its relative bioavailability with the conventional free-drug combination, mapped the spatiotemporal distribution in eight major tissues (including heart, liver, spleen, etc.), and clarified the excretion kinetic characteristics of the TMP NPs/SMZ formulation. In parallel, the synergistic antibacterial activity was confirmed in vitro. Finally, therapeutic efficacy and biosafety were evaluated using a murine *E. coli* infection model. This involved assessing serum biochemistry, quantifying bacterial loads in organs, and analyzing histopathology alongside. Collectively, the findings of this study will provide a solid theoretical basis and experimental data support for the nano-formulation strategy of the classic TMP/SMZ combination.

## 2. Results

### 2.1. Validation of the UPLC-MS/MS Analytical Method

To facilitate pharmacokinetic analysis, we developed a sensitive and robust UPLC-MS/MS method for quantifying TMP and SMZ in rat plasma, tissues, urine, and feces. Method validation adhered to the US Food and Drug Administration (FDA) and the International Council for Harmonisation of Technical Requirements for Pharmaceuticals for Human Use (ICH) M10 bioanalytical guidelines [17], utilizing protocols established in our previous work and related literature [13,18]. Representative MS/MS product ion spectra for the analytes and IS are illustrated in Figures S1–S4. Optimized multiple reaction monitoring (MRM) transitions were selected based on signal intensity and fragmentation efficiency: *m*/*z* 291.1/261.1 for TMP and 253.9/155.8 for SMZ (Table 1). Specificity assays confirmed the absence of endogenous interference in any matrix at the retention times of the analytes or IS. As summarized in Table 2 and Table 3, the method exhibited excellent linearity (R^2^ ≥ 0.9915) across all concentration ranges. Precision (relative standard deviation, RSD) ranged from 2.3% to 12.8%, and accuracy (relative error, RE) remained within 1.4–13.5%, fully meeting acceptance criteria. Extraction recoveries varied from 87.3% to 114.2%, with matrix effects confined between 85.2% and 117.3%. Stability testing confirmed analyte integrity under room-temperature storage, in the autosampler, and after three freeze–thaw cycles (RSD < 14.7%). Furthermore, dilution integrity assessments showed that samples diluted 30-fold maintained accuracy and precision within ± 15%. These results confirm that the method is accurate, reliable, and suitable for determining TMP and SMZ concentrations in rat plasma, tissues, urine, and feces.

### 2.2. Pharmacokinetics and Oral Bioavailability of TMP NPs/SMZ

**Figure 1:**
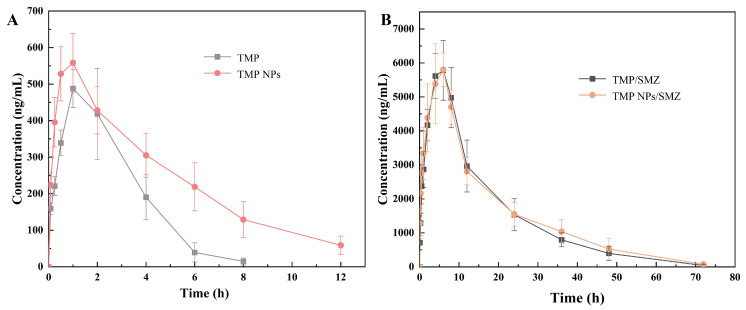
Plasma concentration-time curves of (**A**) TMP and (**B**) SMZ in rats after a single dose oral administration of different TMP/SMZ formulations (TMP, 20 mg/kg; SMZ, 100 mg/kg). For detailed formulation, refer to the Methods Section 4.

TMP has a significantly shorter half-life than its co-administered sulfonamides. This disparity in in vivo elimination rates often necessitates elevated dosages in veterinary practice to ensure therapeutic efficacy. To address this mismatch, we evaluated the pharmacokinetic profiles of TMP NPs versus a conventional TMP formulation following a single oral administration with SMZ in rats. The objective was to determine how the nanoformulation modulates oral absorption and elimination kinetics. Pharmacokinetic parameters and concentration–time profiles are detailed in Tables S1–S4, Table 4 and Table 5, and Figure 1, respectively. Results indicated that TMP NPs exhibited a superior pharmacokinetic profile compared to conventional TMP. While the conventional formulation peaked at 1.33 h and rapidly dropped below 40 ng/mL within 6 h, TMP NPs reached a Cmax of 604.69 ng/mL at 0.83 h and maintained levels above 100 ng/mL for 8 h. Notably, the TMP NPs achieved a relative bioavailability of 193.05%, reflecting substantially enhanced oral absorption. Furthermore, TMP NPs extended the elimination half-life (t_1_/_2_) by 3.37-fold (0.98 h to 3.3 h) and the mean residence time (MRT) from 2.21 h to 3.85 h, accompanied by an increased apparent volume of distribution (Vd) and reduced clearance (CL). As illustrated in Figure 1A, the smoother elimination phase suggests that the nanoformulation effectively retards drug clearance, thereby sustaining therapeutic concentrations [19].

Regarding the co-administered SMZ, no statistically significant differences were observed in AUC, Cmax, or Tmax between the TMP/SMZ and TMP NPs/SMZ groups, nor were there deviations in the concentration-time profiles. This confirms that the nanoformulation selectively improves TMP kinetics without altering SMZ behavior. Consequently, the TMP NPs significantly improved the overlap of effective concentration intervals between the two drugs, narrowing the pharmacokinetic gap and potentially enhancing synergistic efficacy while permitting dosage reduction.

### 2.3. Tissue Distribution Characteristics of TMP NPs/SMZ

**Figure 2:**
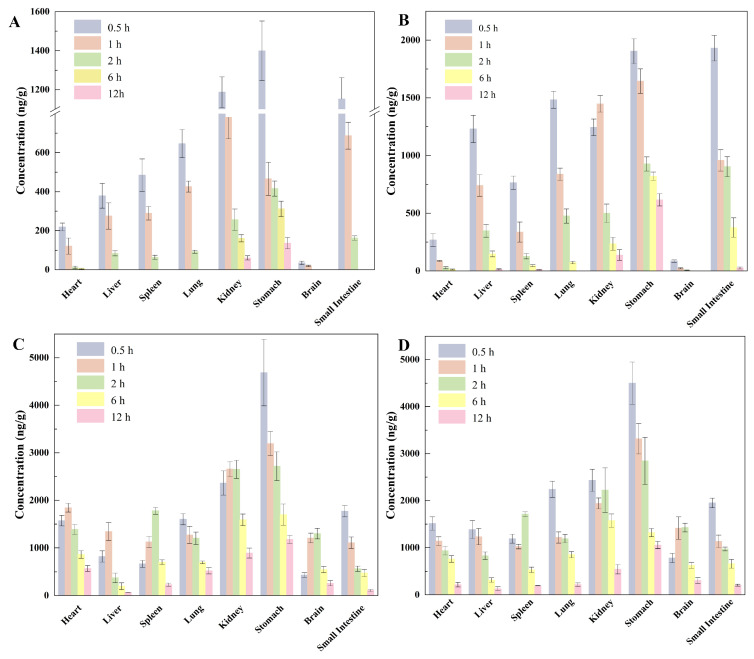
Tissue distribution of TMP and SMZ in rats after a single dose oral administration of different TMP/SMZ formulations (TMP, 20 mg/kg; SMZ, 100 mg/kg). For detailed formulation, refer to the Methods Section 4. (**A**) TMP; (**B**) TMP NPs; (**C**) SMZ in the conventional group; (**D**) SMZ in the nanoparticle group (*n* = 6).

Tables S5 and S6 detail the tissue concentrations of TMP and SMZ following a single oral dose. As shown in Figure 2A,B, the TMP NPs/SMZ group exhibited a tissue distribution profile distinct from the TMP/SMZ group. TMP concentrations in the liver, spleen, lung, kidney, stomach, and small intestine were consistently higher in the nanoparticle group. Crucially, while free TMP was undetectable in the liver, spleen, lung, and small intestine after 6 h, TMP NPs remained measurable in these tissues. Regarding SMZ, tissue distribution was largely comparable between groups (Figure 2C,D and Table S6), mirroring the plasma pharmacokinetic data. Although SMZ levels were higher in the liver, brain, and small intestine for the nanoparticle group, the overall trends were similar, with concentrations maintained at 1000–3000 ng/g in most tissues. In summary, the nanoparticle formulation significantly enhanced TMP tissue exposure and retention without altering SMZ’s biodistribution profile.

### 2.4. Urinary and Fecal Excretion Characteristics of TMP NPs/SMZ

**Figure 3:**
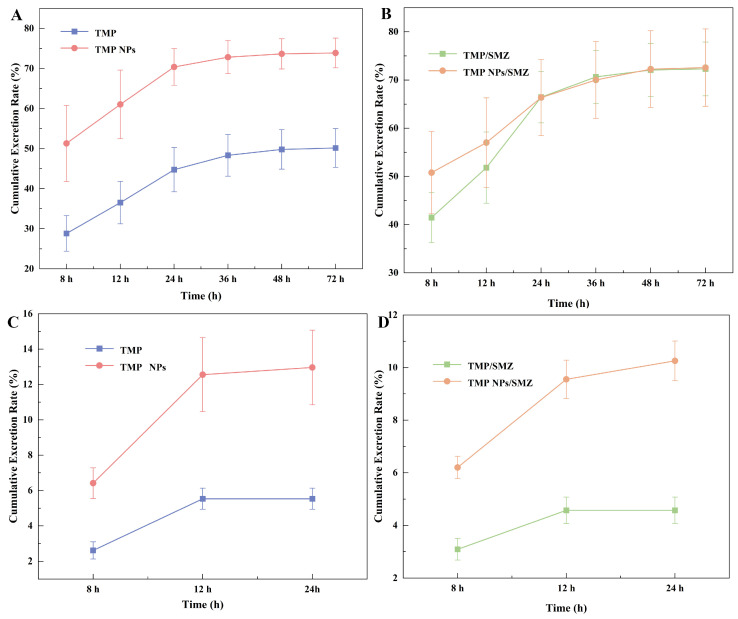
Cumulative urinary (**A**,**B**) and fecal (**C**,**D**) excretion rates of drugs at different time intervals following a single dose oral administration of different TMP/SMZ formulations (TMP, 20 mg/kg; SMZ, 100 mg/kg) in rats (*n* = 6). For detailed formulation, refer to the Methods Section 4.

The excretion rates of TMP and SMZ in urine and feces following a single oral administration are detailed in Tables S7 and S8. Cumulative excretion rates for free TMP and TMP NPs are presented in Table 6 and Figure 3A,C, while co-administered SMZ data are shown in Table 7 and Figure 3B,D. At 72 h post-administration, the cumulative urinary excretion of TMP NPs reached 73.89%, significantly higher than that of free TMP (50.17%). Similarly, SMZ urinary excretion was extensive in both groups (72.32–72.58%). In contrast, fecal excretion was minor: cumulative rates for free TMP and TMP NPs were 5.53% and 12.96%, respectively, with SMZ levels at 4.57% and 10.26%. Regarding excretion kinetics, free TMP and conventional SMZ were undetectable in feces after 24 h, whereas the nanoparticle group showed detectable levels up to 36 h. As indicated in Tables S7 and S8, urinary excretion for both drugs was predominantly concentrated within the first 24 h, followed by a decline until 72 h. Fecal elimination occurred primarily within the first 12 h. Nanoparticle bioadhesion to the gastrointestinal mucosa prolonged drug retention time and, consequently, the fecal excretion phase [20]. For the conventional group, fecal TMP and SMZ levels fell below the detection limit after 12 h. But, the nano-formulation exhibited sustained fecal excretion, with both drugs remaining detectable up to 24 h post-dosing. Calculated from Figure 3A,C, the total recovery (urine + feces) of TMP NPs was 86.85%, compared to 55.7% for free TMP; for SMZ, recovery was 82.84% and 76.89%, respectively. This suggests that the nanocarrier promotes the metabolism and excretion of both TMP and co-administered SMZ.

### 2.5. In Vitro Antibacterial Activity Against E. coli and S. aureus

The in vitro antibacterial activities of SMZ, TMP, TMP NPs, and their combinations against *E. coli* and *S. aureus* were assessed via broth microdilution (Table 8). SMZ exhibited an MIC of 256 µg/mL against both strains, whereas free TMP showed MICs of 1 µg/mL and 8 µg/mL against *E. coli* and *S. aureus*, respectively. These values align with CLSI protocols, ensuring assay validity. Notably, TMP NPs lowered the MICs to 0.5 µg/mL and 2 µg/mL, representing a 2- to 4-fold increase in potency compared to free TMP. Similarly, the TMP NPs/SMZ formulation achieved MICs of 0.625/0.125 µg/mL, a 4-fold improvement over the conventional TMP/SMZ combination. These data suggested that nanoencapsulation not only boosts the intrinsic activity of TMP but also amplifies its synergistic efficacy when co-administered with SMZ. Checkerboard assays further elucidated the interactions between TMP formulations and SMZ (Table 9). For *E. coli*, both TMP/SMZ (FICI = 0.375) and TMP NPs/SMZ (FICI = 0.3125) displayed synergistic effects, consistent with known sulfonamide-TMP interaction profiles [21]. In the case of *S. aureus*, the conventional TMP/SMZ combination showed an additive effect (FICI = 0.5625). However, the TMP NPs/SMZ group achieved an FICI of 0.5, fulfilling the criteria for synergy. These results suggest that nanoencapsulation not only enhances the efficacy of TMP itself but also shifts the TMP/SMZ interaction against *S. aureus* from additive to synergistic.

**Figure 4:**
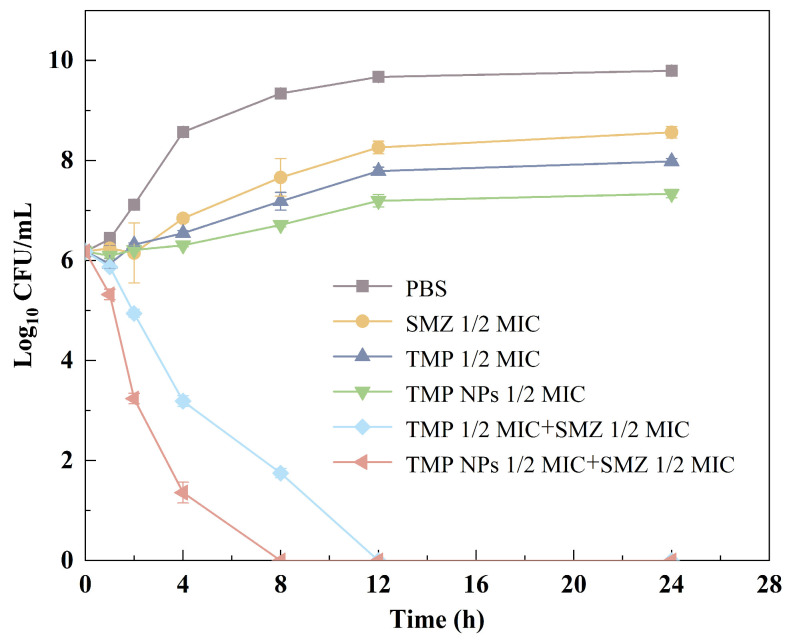
Time–kill curves of different drug combinations against *E. coli*.

Time–kill assays characterized the bactericidal dynamics of the formulations against *E. coli* (Figure 4). Monotherapy with TMP, TMP NPs, or SMZ resulted in a transient reduction in colony counts within the first 12 h, indicating a bacteriostatic phase followed by bacterial regrowth. In contrast, both combination regimens achieved rapid sterilization. Notably, the TMP NPs/SMZ combination eradicated *E. coli* within 8 h, outpacing the free TMP/SMZ group, which required 12 h. These kinetic profiles confirm that the nano-formulation not only retains antibacterial potency but also accelerates bacterial clearance, validating its role as a potentiator.

### 2.6. In Vivo Efficacy and Safety Against E. coli Infection

**Figure 5:**
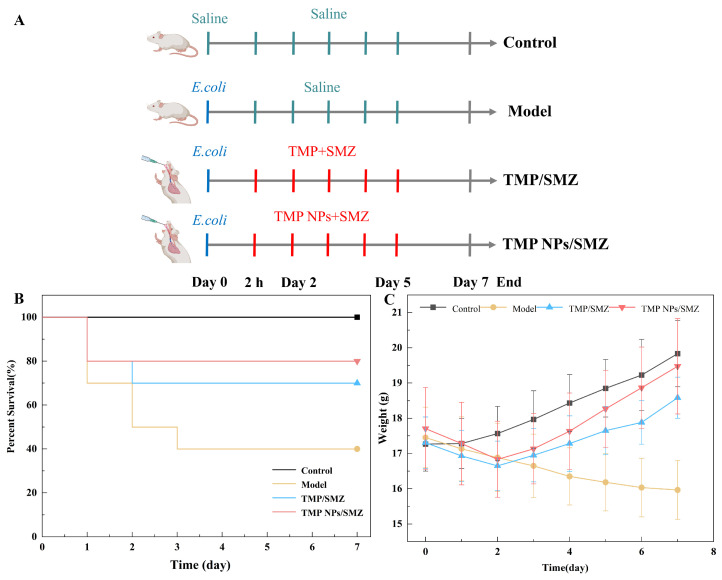
(**A**) Schematic diagram of experimental grouping and procedure for oral administration in *E. coli*-infected mice. (**B**) Survival rates of mice (*n* = 10). (**C**) Body weight changes of mice (*n* = 10). The Model and Control groups were administered an equivalent volume of normal saline.

To evaluate the in vivo therapeutic efficacy of TMP NPs/SMZ, we established an intraperitoneal *E. coli* infection model as outlined in Figure 5A. Mice received a lethal dose (LD_50_) challenge followed by oral treatment 2 h post-infection. The validity of the model was confirmed by the rapid onset of clinical symptoms in infected groups, including lethargy, ruffled fur, hypolocomotion, and anorexia, alongside significant weight loss and mortality within 24–48 h. As shown in Figure 5B,C, the control group remained healthy, exhibiting 100% survival and steady weight gain. In contrast, the untreated model group suffered a continuous decline in body weight and reached a survival rate of only 40%. Treatment intervention significantly improved outcomes: the TMP NPs/SMZ group achieved an 80% survival rate, surpassing the 70% observed in the TMP/SMZ group. While both treatment groups began to regain weight by day 2, the recovery was more rapid and pronounced in mice treated with the nano-formulation. Consequently, TMP NPs/SMZ demonstrated superior antibacterial activity over the conventional formulation, effectively reducing mortality and accelerating physiological recovery.

Serum biochemical analysis confirmed that *E. coli* infection induced acute hepatic and renal impairment. Compared to the blank control, the model group exhibited significantly elevated levels of total protein (TP), alanine aminotransferase (ALT), aspartate aminotransferase (AST), and creatinine (CR). Conversely, alkaline phosphatase (ALP), albumin (ALB), and triglyceride (TG) levels remained unchanged, pinpointing the organ damage specifically to the liver and kidneys [22] (Table 10). Regarding treatment efficacy, the TMP/SMZ group showed reduced ALT, AST, and CR levels compared to the model group; however, ALT levels remained significantly above baseline, and a concurrent spike in TG was observed. This suggests that while free TMP/SMZ mitigated organ dysfunction, it failed to fully reverse the damage. By comparison, the TMP NPs/SMZ group displayed biochemical profiles comparable to the blank control, with no statistically significant differences across all indices (except for a minor TG elevation). These results indicate that TMP NPs/SMZ offer superior protection against infection-induced organ damage, effectively restoring hepatic and renal markers to normal levels while maintaining a favorable safety profile.

**Figure 6:**
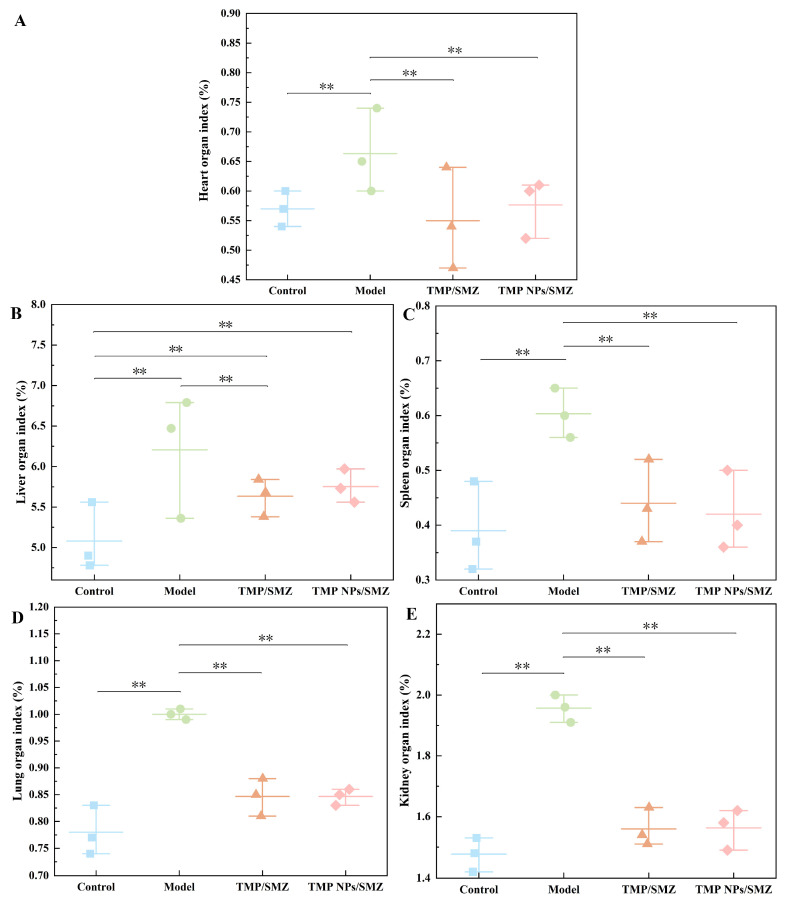
Organ coefficients of mice across different treatment groups. (**A**) Heart, (**B**) liver, (**C**) spleen, (**D**) lung, and (**E**) kidney (*n* = 3). ** *p* < 0.01.

Organ coefficients are widely used biological indicators for evaluating visceral congestion and swelling. Figure 6 illustrates the organ coefficients for the heart, liver, spleen, lung, and kidney across treatment groups. Compared with the control, the TMP/SMZ and TMP NPs/SMZ groups showed significant deviations only in liver coefficients, with no statistical differences observed in the other 4 organs. Conversely, the model group exhibited broadly elevated coefficients, significantly distinguishing it from the other three groups and suggesting pathological alterations, such as hyperplasia or edema [23]. Most importantly, the lack of significant differences between the treatment groups (TMP/SMZ, TMP NPs/SMZ) and the control group indicates that prolonged oral administration of these formulations did not induce gross pathological toxicity in visceral organs.

**Figure 7:**
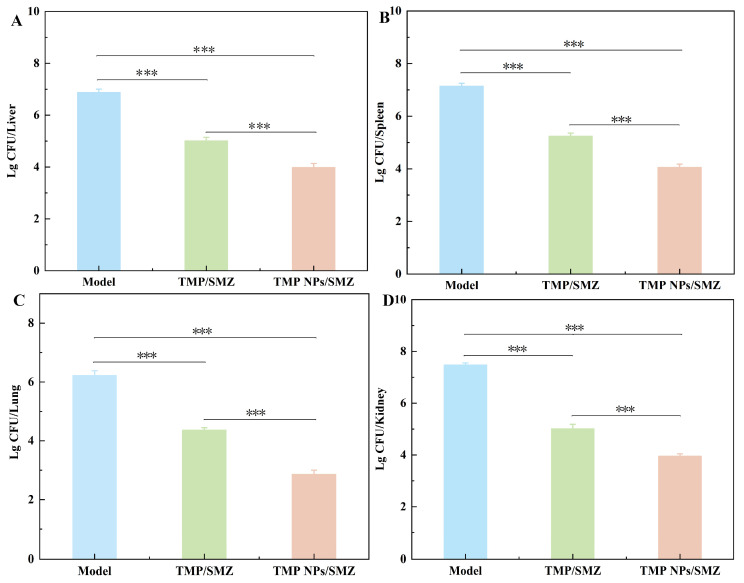
Bacterial loads in tissues of mice across different treatment groups. (**A**) Liver, (**B**) spleen, (**C**) lung, and (**D**) kidney (*n* = 3). *** *p* < 0.001.

Following treatment, tissues (liver, spleen, lung, and kidney) were homogenized to quantify bacterial burdens via plate counts (Figure 7). Both combination therapies achieved substantial bacterial clearance compared to the model group (*p* < 0.001). Notably, the TMP NPs/SMZ formulation demonstrated superior efficacy over the free TMP/SMZ group, further reducing Lg CFU values by 1.02 (liver), 1.19 (spleen), 1.51 (lung), and 1.06 (kidney). These statistically significant reductions (*p* < 0.001) confirm that the nanoparticle system more markedly alleviated bacterial burdens in the liver, spleen, lung, and kidney of infected mice compared with TMP/SMZ.

**Figure 8:**
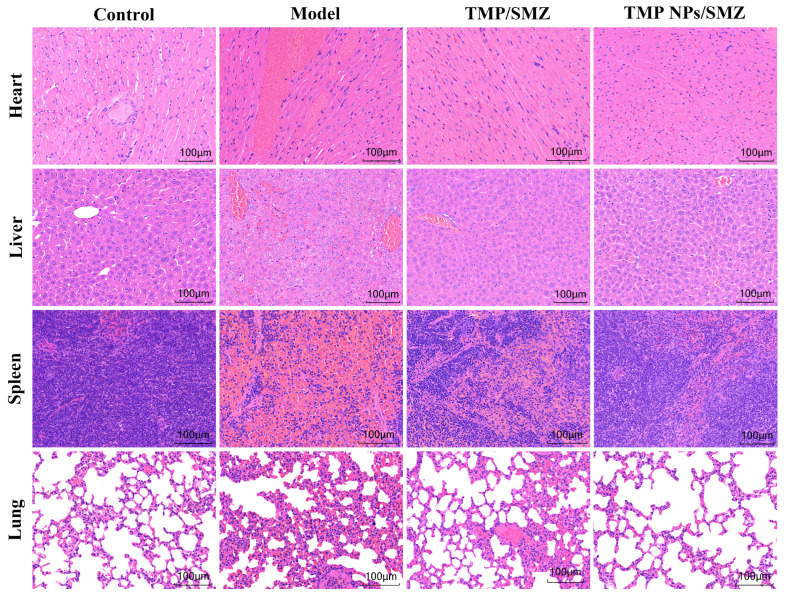
Histopathological sections of heart, liver, spleen, and lung tissues from different groups of mice.

Histological assessment (Figure 8) confirmed that mice in the control group maintained normal cellular morphology and intact tissue architecture, with no discernible lesions. Conversely, the model group exhibited profound pathological disruption across all visceral organs, characterized by cardiomyocyte disarray, hepatic inflammatory infiltration, splenic congestion, and alveolar structural collapse. These features confirm the severity of systemic injury induced by *E. coli*. While TMP/SMZ treatment attenuated these lesions compared with the model group, residual inflammation and morphological irregularities remained evident. In contrast, the TMP NPs/SMZ group showed tissue integrity comparable to that of the control group, with only negligible histological changes. The markedly reduced tissue damage in the nanoparticle-treated group compared with the free drug formulation underscores the superior therapeutic efficacy of TMP NPs/SMZ in preserving visceral organ structure during infection.

## 3. Discussion

The clinical utility of the classic TMP/SMZ combination in veterinary medicine is frequently hindered by TMP’s poor aqueous solubility and the significant difference in their elimination rates. Our findings demonstrate that the PEG-PLGA nanodelivery system effectively bridges this pharmacokinetic gap. The enhanced oral bioavailability (193.05%) and prolonged half-life of TMP are primarily attributable to the physicochemical and biological attributes of the carrier. Specifically, the mucoadhesive and penetration-enhancing properties of PLGA facilitate trans-epithelial transport, directly mitigating the absorption barriers of TMP [24]. Furthermore, the PEG corona provides steric hindrance, reducing immunogenicity and minimizing nonspecific clearance by the mononuclear phagocyte system (MPS) [25,26]. Consequently, the steady-state degradation of the polymeric matrix enables a controlled release profile, effectively synchronizing the in vivo presence of TMP with SMZ to optimize their overlapping therapeutic windows. The tissue distribution profile further reflects the nanocarrier’s systemic behavior, which is predominantly governed by hemodynamics and physiological barriers. The pronounced accumulation of TMP NPs in the liver, spleen, and kidneys aligns with the characteristic perfusion-dependent distribution of polymeric nanoparticles and their passive uptake by the reticuloendothelial system (RES) [27]. Such distribution aligns with established findings that PLGA-based NPs tend to accumulate in these organs [28,29]. Importantly, the enhanced drug exposure and prolonged retention in the lungs and small intestine underscore the specific clinical potential of this nano-formulation for treating respiratory and gastrointestinal infections. Conversely, the negligible brain uptake confirms that PEG-PLGA encapsulation does not alter blood–brain barrier permeability, thereby minimizing the risk of central nervous system toxicity [30]. Elimination kinetics closely mirrored the tissue distribution patterns, with renal clearance established as the primary excretion route. Such excretion behavior is driven by both the intrinsic physicochemical properties of the drugs and those of the PEG-PLGA carrier, as NPs based on this polymer are known to facilitate urinary elimination [31]. The significantly elevated urinary drug concentrations in the nanoparticle group offer a distinct advantage for combating urinary tract infections caused by uropathogens such as *E. coli* or *Streptococcus* [32]. Furthermore, consistent with the mucoadhesive nature of the carrier, the fecal excretion phase was considerably prolonged. The high total recovery rate (86.85%) verifies both the excellent oral absorption efficiency and the absence of irreversible long-term tissue retention, confirming the nanocarrier’s favorable metabolic profile.

Synthesizing the MIC, FICI, and time-kill kinetic data, the TMP NPs markedly amplified both the intrinsic potency of TMP and its synergistic efficacy with SMZ. Two mechanisms appear central to this potentiation: first, the polymeric matrix facilitates membrane penetration and shields the drug from plasma protein binding; second, the potential cationic surface charge may induce electrostatic disruption of bacterial membranes, creating a physical damage effect that synergizes with the encapsulated drug [33,34]. Interestingly, SMZ monotherapy exhibited an identical, relatively high MIC (256 μg/mL) against both Gram-negative *E. coli* and Gram-positive *S. aureus*. This uniform lack of susceptibility across divergent bacterial classes can be attributed to the broad-spectrum mechanism of sulfonamides, which competitively inhibit dihydropteroate synthase, an enzyme highly conserved in the folic acid synthesis pathway across these bacteria [35]. This intrinsically high MIC perfectly illustrates the limited efficacy of SMZ as monotherapy, strongly underscoring the critical need to combine it with TMP to achieve sequential blockade of folate synthesis and the subsequent potent synergistic bactericidal effect. The superior in vitro synergistic activity of the nanoformulation successfully translated into enhanced in vivo therapeutic efficacy in the acute *E. coli* infection model. While the conventional TMP/SMZ formulation provided moderate protection, the TMP NPs/SMZ regimen demonstrated a pronounced clinical advantage, achieving the highest survival rate (80%) and accelerating physiological recovery. This enhanced systemic efficacy is directly correlated with a profound reduction in bacterial burdens across critical organs, particularly the liver, spleen, lungs, and kidneys [36]. Beyond bacterial clearance, the nanoformulation provided superior protection against infection-induced multi-organ dysfunction. Serum biochemical analyses and histological assessments confirmed that while the free TMP/SMZ treatment left residual inflammation and elevated liver enzymes (ALT), the TMP NPs/SMZ regimen effectively restored hepatic and renal markers (ALT, AST, CR) to baseline levels and preserved intact visceral tissue architecture. Regarding in vivo biosafety and tolerability, only a transient, mild elevation in triglycerides (TG) and a slight increase in the liver organ coefficient were observed. As commonly reported with polymeric nanodelivery systems, these minor deviations do not constitute overt systemic toxicity. Rather, they represent a temporary metabolic response to PLGA carrier degradation and subsequent RES accumulation in the liver [37].

Despite the promising results, this study presents certain limitations that warrant further investigation. First, therapeutic efficacy was evaluated exclusively in an acute, single-strain (*E. coli*) murine infection model, which may not fully recapitulate the complexity of polymicrobial or chronic clinical infections. Second, the current experimental design did not include an in vivo efficacy comparison between the TMP NPs/SMZ formulation and other distinct classes of commercial antibiotics. Although our data robustly demonstrate superiority over conventional TMP/SMZ, broader comparative studies against current first-line treatments are indispensable to drive this formulation toward clinical application. Therefore, future research will focus on evaluating the pharmacokinetics and efficacy of this nanodelivery system in target animal models, alongside comprehensive comparisons with various commercial antibiotics. Such subsequent studies will be pivotal in accelerating the clinical translation of this revitalized classic antibiotic combination.

## 4. Materials and Methods

### 4.1. Materials

TMP (CAS: 738-70-5, >98.0%) was purchased from Tokyo Chemical Industry Co., Ltd. (TCI, Tokyo, Japan), while Ormetoprim (OMP) (CAS: 6981-18-6, >99.0%) was obtained from SCR-BIOTECH (Shanghai, China). The sulfonamides, SMZ (CAS: 723-46-6, >99.7%) and Sulfamonomethoxine (SMM) (CAS: 1220-83-3, ≥95.0%), were supplied by MedChemExpress (New Jersey, USA) and Solarbio (Beijing, China), respectively. The polymers, including poly(D,L-lactide-co-glycolide) (PLGA, CAS: 26780-50-7, Resomer^®^ RG 504H, 50:50 lactide:glycolide, acid-terminated, Mw 38,000–54,000), polyethylene glycol (PEG, CAS: 25322-68-3, Mw 5500–7500), and polyvinyl alcohol (PVA, CAS: 9002-89-5, Mw 13,000–23,000), were supplied by Sigma-Aldrich (St. Louis, MO, USA). Dichloromethane (DCM, CAS: 75-09-2) was purchased from Tianjin Xinbote Chemical Industry Co., Ltd. (Tianjin, China), while LC-MS grade methanol (MeOH, CAS: 67-56-1) and formic acid (CAS: 64-18-6) were obtained from Fisher Chemicals (Hampton, NH, USA). Ultrapure water prepared via a Milli-Q system (MilliporeSigma, Burlington, MA, USA) was used throughout the study, and all other reagents were of analytical grade.

### 4.2. Animals

Male Sprague-Dawley (SD) rats (180 ± 10 g) and BALB/c mice (6–8 weeks, 18 ± 2 g, equal sex distribution) were employed for the pharmacokinetic and pharmacodynamic studies, respectively. All animals were obtained from the Laboratory Animal Center, Lanzhou Veterinary Research Institute, Chinese Academy of Agricultural Sciences (CAAS).

### 4.3. Preparation of TMP NPs/SMZ

PEG-PLGA NPs loaded with TMP (TMP NPs) were fabricated using a single-emulsion (O/W) solvent evaporation technique, essentially as described in our previous report [13], with minor modifications. Briefly, the organic phase was prepared by dissolving TMP (39 mg), PEG (97.5 mg), and PLGA (390 mg) in DCM. To ensure uniform dispersion, the TMP and PEG solutions were initially mixed by probe sonication (300 W, 4 s on/2 s off) for 1 min, then incorporated into the PLGA solution under magnetic stirring in an ice bath. This organic mixture was subsequently emulsified into 10 mL of aqueous PVA solution (0.5% *w*/*v*) using intermittent probe sonication (300 W) for 5 min under ice cooling. Following emulsification, the organic solvent was removed by vacuum evaporation at 37 °C for 10 min. The resulting NPs were recovered by centrifugation (20,000 rpm, 30 min, 4 °C), washed three times with distilled water to remove unencapsulated drug, and finally lyophilized for 24 h after redispersion and freezing at −80 °C.

The conventional TMP/SMZ formulation was prepared as a physical mixture of unmodified TMP and SMZ at a standard 1:5 mass ratio. For the nano-formulation (TMP NPs/SMZ), the free drug was substituted with the synthesized TMP NPs, while maintaining the identical 1:5 ratio to ensure comparability.

### 4.4. Ultra High Performance Liquid Chromatography–Tandem Mass Spectrometry (UPLC-MS/MS) Analysis

#### 4.4.1. Instruments

UPLC-MS/MS analysis was performed on an AB Sciex ExionLC™ system coupled to a Triple Quad™ 3500 mass spectrometer (Framingham, MA, USA). Chromatographic separation was achieved using an Agilent (Newport, DE, USA) InfinityLab Poroshell 120 EC-C18 column (4.6 × 100 mm, 2.7 µm). Sciex OS 1.7 and Analyst 1.7.1 software were utilized for data acquisition and processing, respectively.

#### 4.4.2. Detection Conditions

The column temperature was maintained at 30 °C. The mobile phase consisted of water (A) and methanol (B), both acidified with 0.1% formic acid, following the gradient profile in Table S9. Flow rate and injection volume were set to 0.5 mL/min and 5 μL, respectively. Mass spectrometric detection was operated in positive electrospray ionization (ESI) mode using multiple reaction monitoring (MRM). Source parameters included: IonSpray voltage 5500 V, temperature 550 °C, curtain gas 35 psi, and both nebulizer (GS1) and auxiliary (GS2) gases at 55 psi (dwell time: 100 ms).

### 4.5. Preparation of Oral Drug Formulations

For the conventional drug combination, appropriate amounts of TMP and SMZ raw powders were weighed. TMP was directly dissolved in PBS (pH 6.8) containing 0.5% Tween 80. SMZ was initially dissolved in a minimal volume of 0.1 mol/L HCl, heated and stirred in a 40 °C water bath until completely clear, and subsequently diluted with the aforementioned PBS. The TMP and SMZ solutions were then mixed to achieve a final oral gavage solution with a TMP concentration of 2 mg/mL and a SMZ concentration of 10 mg/mL.

For the NPs formulation, appropriate amounts of lyophilized PEG-PLGA/TMP NPs and SMZ raw powder were prepared. The PEG-PLGA/TMP NPs were ultrasonically dispersed in normal saline and vortexed. The dissolution and dilution of SMZ were performed using the exact same procedure as described for the conventional group. The two preparations were thoroughly mixed to achieve a final gavage solution with a TMP equivalent concentration of 2 mg/mL and a SMZ concentration of 10 mg/mL.

### 4.6. Pharmacokinetics Evaluations

#### 4.6.1. Oral Bioavailability

Prior to the pharmacokinetic study, twelve male SD rats were fasted for 12 h but maintained free access to water. The animals were randomized into two equal groups (n = 6). The first group received a single oral gavage of TMP/SMZ, while the second group received the TMP NPs/SMZ formulation. For both treatment arms, the dosage was standardized to 20 mg/kg for TMP (or TMP equivalent in NPs) and 100 mg/kg for SMZ. Serial blood samples (500 μL) were drawn from the retro-orbital plexus before administration (0 min) and subsequently at 5, 15, and 30 min, and 1, 2, 4, 6, 8, 12, 24, 36, 48, and 72 h post-dosing. Samples were immediately transferred into heparinized tubes and centrifuged at 3500 rpm for 10 min. The resulting supernatant plasma was collected and stored at −80 °C until quantitative analysis.

To an aliquot of 100 μL plasma, 12.5 μL of each internal standard (IS) working solution (containing 2 μg/mL OMP for TMP and 2 μg/mL SMM for SMZ) was added sequentially. The samples were vortex-mixed thoroughly to achieve a homogeneous mixture. Subsequently, protein precipitation was induced by adding 375 μL of acetonitrile, followed by vortexing for 1 min. The precipitated samples were centrifuged at 12,000 rpm for 10 min to clarify the supernatant. The resulting organic layer was transferred to a fresh tube and evaporated to dryness under vacuum at 350 Pa and 40 °C. This drying process was maintained for approximately 10 h using a sample concentrator to ensure complete solvent removal. Finally, the dried residue was reconstituted in 200 μL of the initial mobile phase, composed of 0.1% formic acid in water and methanol (80:20, *v*/*v*). After filtration through a 0.22 μm membrane, a 5 μL volume of the processed sample was injected into the LC–MS/MS system.

#### 4.6.2. Tissue Distribution

Animal allocation and dosing regimens followed the procedures previously established in Section 4.6.1. Terminal sampling was conducted at 0.5, 1, 2, 6, and 12 h after administration. At each interval, animals were subjected to 3% isoflurane anesthesia prior to euthanasia. Major organs, specifically the heart, liver, spleen, lung, kidney, stomach, brain, and small intestine, were rapidly excised. To ensure sample purity, tissues were rinsed with normal saline to remove adhering blood and luminal contents, then blotted dry using filter paper. After being weighed and recorded, the samples were immediately transferred to −80 °C for storage until analysis [38].

Tissue samples were processed by weighing 0.5 g of the respective organ and combining it with physiological saline at a 1:2 (*w*/*v*) ratio. Homogenization was facilitated by the addition of steel grinding beads. The resulting mixture was subjected to ultrasonication for 5 min, followed by centrifugation at 5000 rpm for 5 min. The upper tissue homogenate layer was aspirated for extraction. An aliquot of 100 μL of this homogenate was spiked with 12.5 μL of the IS working solutions containing OMP (2 μg/mL for TMP) and SMM (2 μg/mL for SMZ). After vortex-mixing, protein precipitation was induced by adding 375 μL of acetonitrile. The samples were vortexed for 1 min and centrifuged at 12,000 rpm for 10 min. The resulting supernatant was transferred to a fresh tube and evaporated to dryness under vacuum (350 Pa) at 40 °C. This drying step was maintained for 10 h to guarantee complete solvent removal. The dried residue was then reconstituted in 200 μL of the mobile phase (0.1% formic acid in water and methanol, 80:20, *v*/*v*), filtered through a 0.22 μm membrane, and 5 μL was injected into the LC–MS/MS system.

#### 4.6.3. Urinary and Fecal Excretion

Twelve rats were individually accommodated in metabolic cages to separate urine and feces. The rats were randomly divided into two equal groups (n = 6), and drug administration was performed as described in Section 4.6.1. Post-administration, urine and feces were harvested separately at scheduled time points covering 0–4, 4–8, 8–12, 12–24, 24–36, 36–48, and 48–72 h. For each interval, urine output was quantified. The urine volume of each time interval was recorded, and the fecal samples were weighed after drying to constant weight. All samples were stored at −80 °C until further analysis [39].

Urine aliquots (100 μL) were spiked with 12.5 μL each of OMP and SMM IS working solutions (2 μg/mL). Liquid–liquid extraction was performed by adding 375 μL of ethyl acetate:chloroform (3:7, *v*/*v*) [40]. The mixture was vortexed for 1 min and centrifuged at 12,000 rpm for 10 min. The supernatant was collected and evaporated to dryness under vacuum (350 Pa) at 40 °C for 10 h. Residues were reconstituted in 200 μL of the initial mobile phase (aqueous formic acid:methanolic formic acid, 80:20 *v*/*v*). After filtration through a 0.22 μm membrane, 5 μL was injected into the LC–MS/MS system.

Fecal samples (0.1 g) were weighed and homogenized with physiological saline (1:10, *w*/*v*) using steel beads. A 100 μL aliquot of the homogenate was spiked with 12.5 μL each of the OMP and SMM IS working solutions (2 μg/mL). Proteins were precipitated using 875 μL of acetonitrile:ethyl acetate (3:7, *v*/*v*) followed by vortex mixing. Subsequent processing steps, including centrifugation, evaporation, and reconstitution, were identical to those described for urine samples.

### 4.7. Antimicrobial Activity Assays

#### 4.7.1. Bacterial Culture

Reference strains sensitive to the TMP/SMZ synergistic combination, specifically *E. coli* ATCC 25922 and *S. aureus* ATCC 29213, were utilized. Frozen stocks were revived by streaking *E. coli* onto LB agar and *S. aureus* onto TSA plates, which were then incubated inverted at 37 °C for 18–24 h. Then, typical single colonies were picked and inoculated into 50 mL centrifuge tubes containing 20 mL of LB broth (for *E. coli*) or TSB broth (for *S. aureus*). The cultures were incubated at 37 °C with constant shaking at 200 rpm for 12 h. The resulting bacterial cultures were adjusted with Mueller–Hinton broth (MHB) to a turbidity equivalent to a 0.5 McFarland standard (approximately 1.5 × 10^8^ CFU/mL). For in vitro antibacterial assays, these standardized suspensions were further diluted with MHB to a final concentration of 1.5 × 10^6^ CFU/mL. For the in vivo infection model, the bacterial cells from the standardized suspension were harvested by centrifugation at 3500 rpm for 10 min. After discarding the supernatant, the pellets were resuspended in sterile normal saline to achieve a concentrated density of 5 × 10^9^ CFU/mL for animal inoculation.

#### 4.7.2. Minimum Inhibitory Concentration (MIC) Assays

The MIC values for TMP NPs/SMZ were determined by the broth microdilution method, following Clinical and Laboratory Standards Institute (CLSI) guidelines [41]. MHB (100 µL) was dispensed into 96-well microplates, followed by the preparation of two-fold serial dilutions for TMP, SMZ, TMP NPs, TMP/SMZ, and TMP NPs/SMZ. Bacterial suspensions (100 µL, 1.5 × 10^6^ CFU/mL) were subsequently inoculated to yield the following final concentration ranges: SMZ (0.5–256 µg/mL), TMP and TMP NPs (0.0625–32 µg/mL), and the combined formulations (0.0625/0.3125–32/160 µg/mL). Sterility (200 µL sterile MHB) and growth controls (100 µL MHB with bacteria) were included in each run. All assays were performed in triplicate. Following incubation at 37 °C for 18–24 h, MIC was defined as the lowest concentration that prevented visible bacterial growth.

#### 4.7.3. Fractional Inhibitory Concentration Index (FICI) Study

The fractional inhibitory concentration index (FICI) regarding the combination of SMZ and TMP or TMP NPs was determined using the checkerboard dilution method. In the assay, 100 µL of MHB was dispensed into each well of a 96-well microtiter plate. SMZ was introduced into the first row at 4 MIC (100 µL) and diluted two-fold vertically down to the seventh row. Subsequently, TMP or TMP NPs (4MIC) were added to the first column to reach a volume of 200 µL, followed by a two-fold horizontal serial dilution across to the ninth column. Finally, 100 µL of bacterial suspension (1.5 × 10^6^ CFU/mL) was inoculated into each well, yielding a final volume of 200 µL. Control wells were designated as follows: SMZ monotherapy (column 10), TMP or TMP NPs monotherapy (row 8), negative control (column 11, MHB only), and positive control (column 12, MHB with bacteria). After incubation at 37 °C for 18–24 h, turbidity was assessed to determine MIC values. The FICI was calculated as:

FICI = MIC_A+B_/MIC_A_ + MIC_B+A_/MIC_B_

Here, MIC_A+B_ and MIC_B+A_ denote the MIC of drugs A and B in combination, while MIC_A_ and MIC_B_ denote their respective monotherapy MICs. Interactions were categorized as: synergism (FICI ≤ 0.5), addition (0.5 < FICI ≤ 1), indifference (1 < FICI ≤ 4), or antagonism (FICI > 4) [42].

#### 4.7.4. Time–Kill Kinetics Assay

Following the checkerboard assay, time-kill kinetics against *E. coli* were evaluated by colony counting to verify the synergism of SMZ with either free TMP or TMP NPs. Experimental arms included a negative control, positive control, monotherapies (free TMP, TMP NPs), and combinations (TMP/SMZ, TMP NPs/SMZ). Bacterial inocula were prepared as detailed in Section 4.7.1, diluted in MHB to 1.5 × 10^6^ CFU/mL. Cultures were then challenged with drugs at 0.5×, 1×, and 2× MIC based on previously determined values. At 0, 1, 2, 4, 8, 12, and 24 h post-exposure, 50 µL aliquots were withdrawn and diluted ten-fold in phosphate-buffered saline (PBS, 450 µL). Subsequently, 50 µL of the diluted suspension was plated onto MHA for enumeration. After incubation at 37 °C for 18–24 h, viable counts were determined. Time-kill profiles were plotted as Log_10_ CFU/mL versus incubation time, with total CFU calculated as follows: CFU= colony count × dilution factor [43].

To ensure the validity of the in vitro antimicrobial activity assay, uninoculated medium was included as a negative sterility control, while drug-free medium (or PBS) inoculated with bacteria served as a positive growth control (vehicle negative control). Furthermore, the conventional free TMP/SMZ combination was employed as the positive drug control to provide a comparative baseline for evaluating the efficacy enhancement of the TMP NPs/SMZ.

### 4.8. In Vivo Pharmacodynamics

#### 4.8.1. Therapeutic Efficacy of Oral TMP NPs/SMZ Against Intraperitoneal *E. coli* Infection

Model establishment: A murine intraperitoneal (i.p.) infection model was established as described by Li et al. [44]. *E. coli* 25922 suspensions, prepared as detailed in Section 4.7.1, were adjusted with sterile saline to produce a concentration gradient (5 × 10^7^, 1 × 10^8^, 5 × 10^8^, 1 × 10^9^, and 5 × 10^9^ CFU/mL). Fifty BALB/c mice (equal gender distribution) were randomized into five groups (n = 10) and injected i.p. with 300 µL of the respective inocula. Mortality was monitored for 5 days; the concentration inducing approximately 50% lethality was defined as the optimal inoculum for subsequent efficacy trials.

In vivo therapeutic efficacy assessment: Forty BALB/c mice (equal gender distribution) were randomized into four arms (n = 10): Control (saline only), Model (untreated infection), and two treatment groups (TMP/SMZ and TMP NPs/SMZ). Infection was induced in the model and treatment groups via i.p. injection of 300 µL bacterial suspension (5 × 10^8^ CFU/mL), while the control group received sterile saline. Treatment was initiated 2 h post-infection via oral gavage. The treatment groups received 20 mg/kg TMP (or TMP NPs) combined with 100 mg/kg SMZ, whereas the control and model groups received saline. Dosing continued once daily for 5 days. To assess bacterial burden, three mice from the model group were aseptically sacrificed at 24 h post-infection, and the liver, spleen, lungs, and kidneys were harvested. Clinical signs, survival rates, and body weights of the remaining mice were recorded daily. The study concluded on day 7; surviving mice were euthanized for necropsy to quantify visceral bacterial loads and calculate organ coefficients.

#### 4.8.2. Serum Biochemical Analysis

Upon completion of the treatment regimen, six mice per group were randomly selected for sampling. Blood was harvested via aseptic orbital enucleation using sterile ophthalmic forceps and transferred into sterile tubes. Samples were allowed to clot at room temperature for 30 min, followed by centrifugation at 3500 rpm for 15 min at 4 °C to isolate serum. The resulting serum was stored at −20 °C pending analysis. Key biochemical markers, including alkaline phosphatase (ALP), albumin (ALB), total protein (TP), alanine aminotransferase (ALT), aspartate aminotransferase (AST), creatinine (CR), and triglycerides (TG), were quantified using an XL-640 automatic biochemical analyzer.

#### 4.8.3. Assessment of Organ Coefficients

To evaluate the impact of the 5-day oral TMP NPs/SMZ regimen on visceral organs, three mice per group were randomly selected for necropsy at the study endpoint. The heart, liver, spleen, lung, and kidneys were harvested, and their wet weights were determined. The organ coefficient (OC) for each tissue was subsequently calculated using the formula [44]:

OC = Organ weight (g)/Body weight (g).

#### 4.8.4. Quantification of Bacterial Burden in Organs

The bacterial load in the liver, spleen, lung, and kidney tissues of mice from the model group, TMP/SMZ group, and TMP NPs/SMZ group was quantified by colony counting. Briefly, 0.1 g of each tissue sample was aseptically harvested and homogenized in sterile normal saline at a weight-to-volume ratio (*w*/*v*) of 1:10. The resulting tissue homogenates were then subjected to 10-fold serial dilution with sterile normal saline. A 50 µL aliquot of each dilution gradient was uniformly spread onto LB agar plates, which were then incubated at 37 °C for 24 h. In healthy mice, the intestinal barrier and mucosal immune system restrict commensals to the gut and mesenteric lymph nodes, preventing significant colonization of deep organs [45]. Thus, the recovered colonies overwhelmingly represented the inoculated *E. coli*. To ensure specificity, the uniformity of colony morphology was visually verified prior to counting to exclude potential polymicrobial contamination. Plates with colony counts ranging from 30 to 300 were selected for bacterial load calculation, with three parallel replicates per group. The bacterial load results were converted to colony-forming unit (CFU) values in the original tissues and expressed as Log_10_ CFU/organ [46].

#### 4.8.5. Histopathological Examination

Following euthanasia, the heart, liver, spleen, and lung were immediately excised, rinsed with sterile saline, and fixed in 4% paraformaldehyde for 24 h. Fixed specimens underwent standard histological processing, including gradient dehydration, clearing, and paraffin embedding. Serial sections were cut, stained with hematoxylin and eosin (H&E), and mounted with neutral balsam. Pathological changes were examined under a light microscope. Therapeutic efficacy was evaluated by comparing the severity and histopathological characteristics across groups.

### 4.9. Statistical Analysis

Quantitative results are expressed as mean ± standard deviation. Statistical analyses were executed using SPSS software (version 27.0, IBM, New York, NY, USA). Differences between the control and experimental groups were assessed using one-way analysis of variance (ANOVA).

## 5. Conclusions

This study systematically validates a PEG-PLGA nanoparticle strategy designed to overcome the inherent biopharmaceutical limitations that compromise the classic synergistic combination of TMP and SMZ. By reformulating TMP into a nano-delivery system, we successfully reshaped its pharmacokinetic profile, achieving a 3.37-fold extension in elimination half-life and a nearly 2-fold increase in oral bioavailability. While not eliminating the kinetic differences entirely, this significant extension of TMP’s systemic circulation effectively bridges the gap with the longer-persisting SMZ, thereby sustaining the synergistic ratio required for optimal sequential blockade of folate metabolism. These pharmacokinetic improvements translated directly into superior therapeutic outcomes. The formulation demonstrated enhanced tissue penetration, particularly in the liver, spleen, and lungs, and maintained detectable concentrations well beyond the free-drug window. In vitro, this resulted in a 2- to 4-fold reduction in MIC against *E. coli* and *S. aureus*, confirming that the carrier preserves and potentially potentiates intrinsic drug activity. More importantly, in a murine infection model, the nano-regimen conferred a distinct survival advantage (80%) and accelerated bacterial clearance compared to conventional therapy. Crucially, regarding biosafety, the nanoparticle formulation alleviated the hepatic and renal stress typically associated with the infection and drug treatment. Histopathological and biochemical assessments confirmed that the therapy effectively prevented severe inflammatory progression and facilitated the return of organ function markers to baseline levels. Collectively, these findings provide a robust rationale for using nanotechnology to revitalize this essential antibiotic combination, offering a promising pathway to enhance efficacy and reduce toxicity in clinical applications.

## Figures and Tables

**Table 1:** Optimized MRM transitions and mass spectrometric parameters (DP, CE) for TMP, SMZ, and their respective internal standards.

Sample	Polarity	Precursor Ion (*m*/*z*)	Product Ion (*m*/*z*)	DP (V)	CE (V)	Retention Time (Minutes)
TMP	+	291.1	261.1 */275.1	109/115	35/36	4.94
OMP	+	275.1	259.1 */231.1	58/60	35/34	5.32
SMZ	+	253.9	155.8 */108.0	70	23/35	5.92
SMM	+	278.9	185.8 */155.9	80	27	5.01

Note: DP: declustering potential; CE: collision energy; *: quantitative ion.

**Table 2:** The limit of detection (LOD), limit of quantification (LOQ), calibration curve, and linear range of TMP in various biological matrices of rats.

Biological Matrices	Calibration Curve	R^2^	Linear Range (ng/mL)	LOQ	LOD
Plasma	Y = 0.00738X + 0.05602	0.99481	1–200	1	0.5
Heart	Y = 0.00470X + 0.04737	0.99153	4–200	4	2
Liver	Y = 0.00684X + 0.05980	0.99692	4–200	4	2
Spleen	Y = 0.00658X + 0.06348	0.99779	4–200	4	2
Lung	Y = 0.00492X + 0.06580	0.99792	4–200	4	2
Kidney	Y = 0.00625X + 0.04123	0.99654	4–200	4	2
Stomach	Y = 0.00435X + 0.05215	0.99188	4–200	4	2
Brain	Y = 0.00518X − 0.00350	0.99321	4–200	4	2
Small Intestine	Y = 0.00610X + 0.02367	0.99590	4–200	4	2
Urine	Y = 0.00764X − 0.01243	0.99742	1–200	1	0.5
Feces	Y = 0.00488X + 0.06853	0.99265	4–200	4	2

**Table 3:** The LOD, LOQ, calibration curve, and linear range of SMZ in various biological matrices of rats.

Biological Matrices	Calibration Curve	R^2^	Linear Range (ng/mL)	LOQ	LOD
Plasma	Y = 0.00837X + 0.18049	0.99673	20–1000	20	5
Heart	Y = 0.00843X + 0.21111	0.99190	20–1000	20	5
Liver	Y = 0.00971X + 0.13288	0.99431	20–1000	20	5
Spleen	Y = 0.01382X + 0.07056	0.99935	20–1000	20	5
Lung	Y = 0.01368X + 0.08146	0.99865	20–1000	20	5
Kidney	Y = 0.01245X + 0.09212	0.99782	20–1000	20	5
Stomach	Y = 0.00986X − 0.00428	0.99478	20–1000	20	5
Brain	Y = 0.00912X − 0.01541	0.99564	20–1000	20	5
Small Intestine	Y = 0.01305X + 0.06545	0.99901	20–1000	20	5
Urine	Y = 0.01058X + 0.14520	0.99321	20–1000	20	5
Feces	Y = 0.01134X + 0.08765	0.99645	20–1000	20	5

**Table 4:** Main pharmacokinetic parameters of TMP in rats following single-dose oral administration of different formulations of TMP combined with SMZ (*n* = 6).

Parameter	Unit	TMP/SMZ	TMP NPs/SMZ
AUC_0–t_	h·ng/mL	1648.33 ± 382.74	2922.17 ± 529.81 **
AUC_0–∞_	h·ng/mL	1672.00 ± 389.30	3227.83 ± 623.52 **
Cmax	ng/mL	526.32 ± 61.46	604.69 ± 50.80
Tmax	h	1.33 ± 0.52	0.83 ± 0.26
t_1/2_	h	0.98 ± 0.13	3.30 ± 0.95 **
MRT	h	2.21 ± 0.25	3.85 ± 0.50 **
Vz	L/kg	17.33 ± 3.49	29.46 ± 6.59 **
CL	L/h/kg	12.48 ± 2.77	6.44 ± 1.56 **
F (%)	/	/	193.05%

Note: ** Statistical significance compared with TMP/SMZ is *p* < 0.01.

**Table 5:** Main pharmacokinetic parameters of SMZ in rats following single-dose oral administration of different formulations of TMP combined with SMZ (*n* = 6).

Parameter	Unit	TMP/SMZ	TMP NPs/SMZ
AUC_0–t_	h·ng/mL	104,992.83 ± 23,262.03	110,182.50 ± 23,766.20
AUC_0–∞_	h·ng/mL	107,316.00 ± 23,117.41	111,545.33 ± 23,794.96
Cmax	ng/mL	6140.73 ± 654.67	6166.84 ± 629.46
Tmax	h	5 ± 1.10	5 ± 1.10
t_1/2_	h	9.44 ± 2.40	9.97 ± 0.88
MRT	h	14.99 ± 2.24	16.58 ± 2.54
Vz	L/kg	12.86 ± 3.00	13.15 ± 1.79
CL	L/h/kg	0.97 ± 0.23	0.93 ± 0.21
F (%)	/	/	105%

**Table 6:** Cumulative urinary and fecal excretion rates of TMP in rats following single-dose oral administration of two TMP/SMZ formulations (*n* = 6).

Matrix	Time Interval (h)	Cumulative Excretion Rate ($\bar{x}$ ± s,%)	
		TMP/SMZ	TMP NPs/SMZ
Urine	0–4	-	-
	4–8	28.83 ± 4.44	51.32 ± 9.50
	8–12	36.54 ± 5.31	61.05 ± 8.56
	12–24	44.76 ± 5.53	70.39 ± 4.57
	24–36	48.35 ± 5.22	72.85 ± 4.13
	36–48	49.82 ± 4.93	73.67 ± 3.79
	48–72	50.17 ± 4.85	73.89 ± 3.71
Feces	0–4	-	-
	4–8	2.61 ± 0.49	6.42 ± 0.87
	8–12	5.53 ± 0.60	12.55 ± 2.09
	12–24	-	12.96 ± 2.11
	24–36	-	-

**Table 7:** Cumulative urinary and fecal excretion rates of SMZ in rats following single-dose oral administration of two TMP/SMZ formulations (*n* = 6).

Matrix	Time Interval (h)	Cumulative Excretion Rate ($\bar{x}$ ± s,%)	
		TMP/SMZ	TMP NPs/SMZ
Urine	0–4	-	-
	4–8	41.47 ± 5.20	50.80 ± 8.54
	8–12	51.82 ± 7.39	57.02 ± 9.28
	12–24	66.46 ± 5.34	66.37 ± 7.88
	24–36	70.65 ± 5.50	70.02 ± 7.96
	36–48	72.08 ± 5.52	72.27 ± 7.95
	48–72	72.32 ± 5.57	72.58 ± 8.03
Feces	0–4	-	-
	4–8	3.09 ± 0.41	6.20 ± 0.43
	8–12	4.57 ± 0.50	9.56 ± 0.73
	12–24	-	10.26 ± 0.75
	24–36	-	-

**Table 8:** MIC values of different treatment groups against *E. coli* and *S. aureus* (µg/mL).

Strains	SMZ	TMP	TMP NPs	TMP/SMZ	TMP NPs/SMZ
*E. coli*	256	1	0.5	2.5/0.5	0.625/0.125
*S. aureus*	256	8	2	2.5/0.5	0.625/0.125

**Table 9:** FICI values of TMP combined with SMZ in different dosage forms against *E. coli* and *S. aureus*.

Strains	TMP/SMZ		TMP NPs/SMZ	
	FICI	Interaction Type	FICI	Interaction Type
*E. coli*	0.375	Synergistic effect	0.3125	Synergistic effect
*S. aureus*	0.5625	Additive effect	0.5	Synergistic effect

**Table 10:** Serum biochemical parameters of mice treated with different TMP/SMZ formulations (*n* = 6).

Detection Index	Unit	Blank Control	Model	TMP/SMZ	TMP NPs/SMZ
ALP	U/L	77.17 ± 7.87	84.50 ± 5.79	75 ± 11.87	72.83 ± 4.45
ALB	g/L	27.50 ± 2.25	31.87 ± 8.68	23.67 ± 1.74	24.15 ± 2.51
TP	g/L	59.08 ± 8.46	73.87 ± 17.58 *	59.20 ± 1.95	60.73 ± 3.50
ALT	U/L	45.13 ± 9.76	106.98 ± 30.74 *	81.80 ± 4.89 *	43.77 ± 9.68
AST	U/L	148.72 ± 25.19	321.95 ± 169.29 *	175.80 ± 16.74	143.90 ± 22.85
CR	µmol/L	14.05 ± 5.04	35.32 ± 8.36 *	9.38 ± 3.02	9.68 ± 2.64
TG	mmol/L	1.01 ± 0.26	0.96 ± 0.12	1.27 ± 0.12 *	1.23 ± 0.13 *

Note: * Statistical significance compared with blank control group is *p* < 0.05.

## Data Availability

The original contributions presented in this study are included in the article/Supplementary Material. Further inquiries can be directed to the corresponding authors.

## Supplementary Materials

The following supporting information can be downloaded at: https://www.mdpi.com/article/10.3390/antibiotics15030283/s1, Figure S1: Representative MS/MS product ion spectrum of TMP; Figure S2: Representative MS/MS product ion spectrum of OMP; Figure S3: Representative MS/MS product ion spectrum of SMZ; Figure S4: Representative MS/MS product ion spectrum of SMM; Table S1: Plasma concentrations of TMP (ng/mL) at various time points after a single oral dose of TMP/SMZ in rats (*n* = 6); Table S2: Plasma concentrations of TMP (ng/mL) at various time points after a single oral dose of TMP NPs/SMZ in rats (*n* = 6); Table S3: Plasma concentrations of SMZ (ng/mL) at various time points after a single oral dose of TMP/SMZ in rats (*n* = 6); Table S4: Plasma concentrations of SMZ (ng/mL) at various time points after a single oral dose of TMP NPs/SMZ in rats (*n* = 6); Table S5: Tissue distribution concentrations of TMP (ng/g) at various time points after single oral dose of two TMP/SMZ formulations in rats (x ± s, *n* = 6); Table S6: Tissue distribution concentrations of SMZ (ng/g) at various time points after single oral dose of two TMP/SMZ formulations in rats (x ± s, *n* = 6); Table S7: Urinary and fecal excretion rates of TMP in rats following single-dose oral administration of two TMP/SMZ formulations (*n* = 6); Table S8: Urinary and fecal excretion rates of SMZ in rats following single-dose oral administration of two TMP/SMZ formulations (*n* = 6), Table S9: Liquid chromatography gradient elution profile, and the application format for ethical approval for research involving animal.
