# Supplementary material for: Revitalizing Trimethoprim/Sulfamethoxazole via Nanotechnology for Improved Pharmacokinetics and Antibacterial Efficacy
Source: Antibiotics (Basel). 2026 Mar 10;15(3):283. doi: 10.3390/antibiotics15030283 (PMC13024217; doi:10.3390/antibiotics15030283)
Supplement: Supplementary file 1 [file antibiotics-15-00283-s001.zip › antibiotics-4142631-supplementary.pdf]

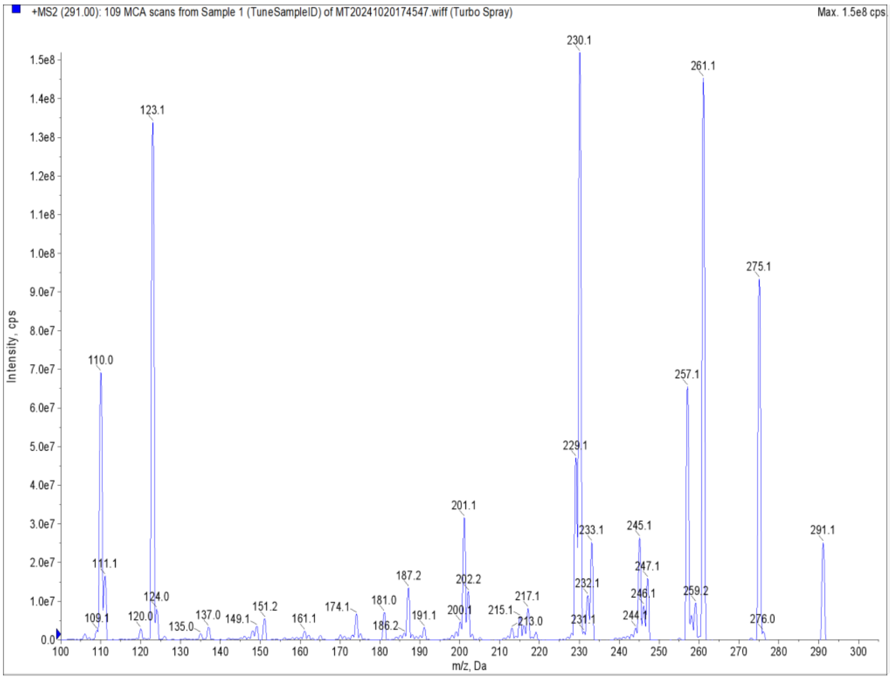

Figure S1. Representative MS/MS product ion spectrum of TMP.

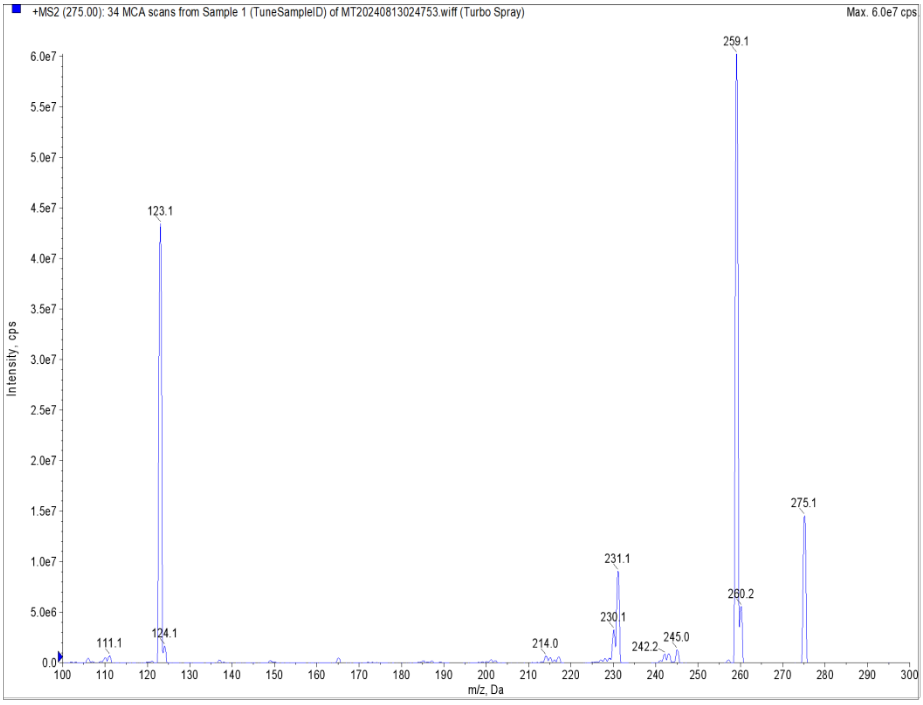

Figure S2. Representative MS/MS product ion spectrum of OMP.

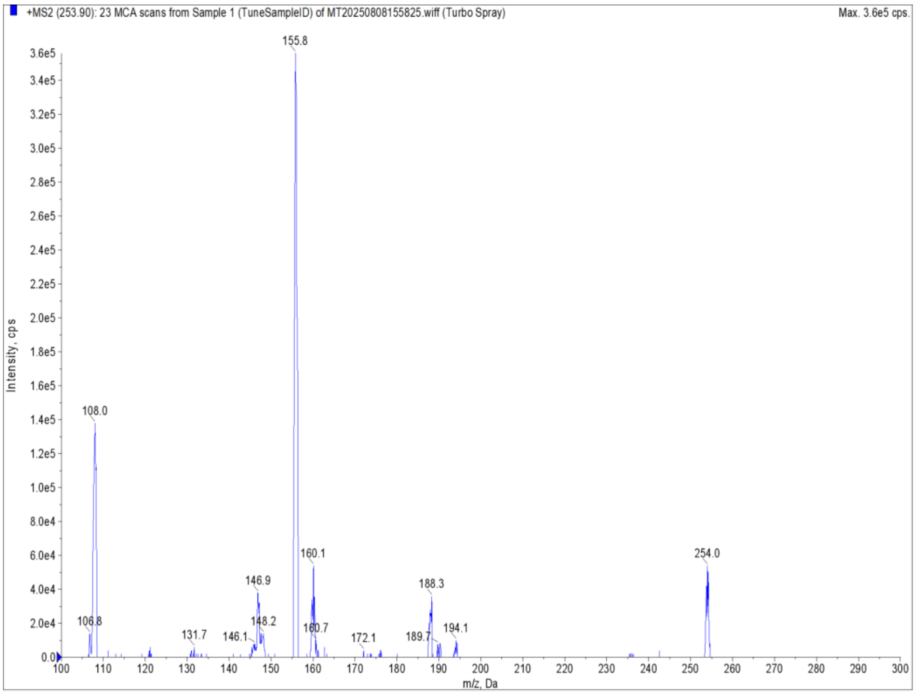

Figure S3. Representative MS/MS product ion spectrum of SMZ.

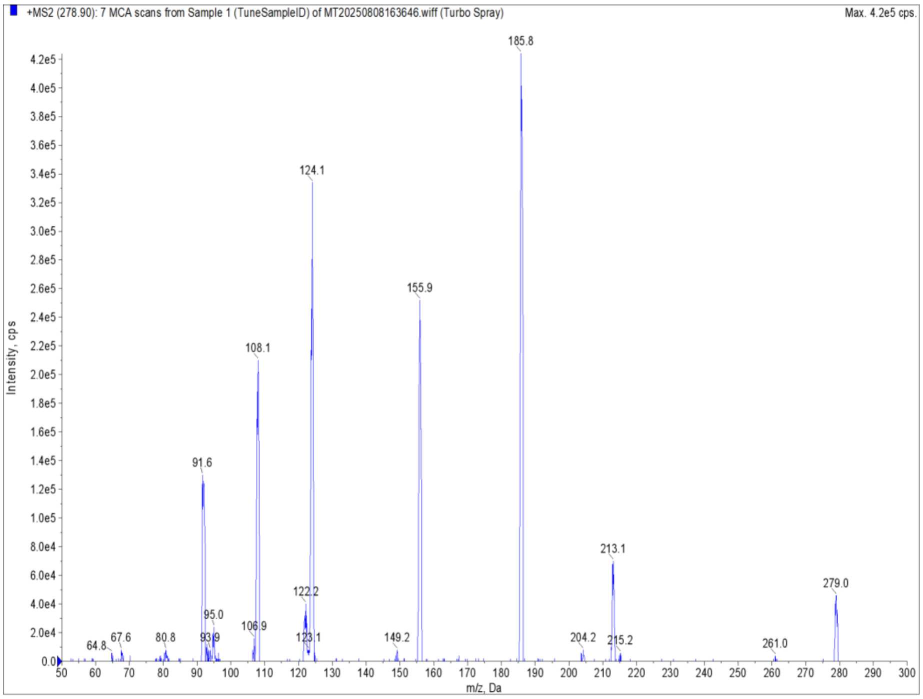

Figure S4. Representative MS/MS product ion spectrum of SMM.

Table S1. Plasma concentrations of TMP (ng/mL) at various time points after a single oral dose of TMP/SMZ in rats (n=6).

time（h）	1	2	3	4	5	6	Mean	SD
0.083	158.49	172.05	139.63	178.19	142.45	163.21	159.00	15.53
0.25	217.88	256.3	199.56	241.58	186.8	222.68	220.80	25.73
0.5	362.63	386.42	304.74	357.65	298.32	324.39	339.03	35.20
1	568.33	453.28	471.9	533.29	433.18	466.37	487.73	51.85
2	358.17	592.61	316.54	372.39	311.68	558.58	418.33	124.51
4	187.56	254.3	130.34	166.37	128.76	273.11	190.07	61.51
6	31.66	65.33	15.47	24.14	20.65	78.38	39.27	26.11
8	8.32	17.98	ND	5.8	ND	26.21	14.58	9.36
12	ND	ND	ND	ND	ND	ND	ND	ND
24	ND	ND	ND	ND	ND	ND	ND	ND
36	ND	ND	ND	ND	ND	ND	ND	ND
48	ND	ND	ND	ND	ND	ND	ND	ND
72	ND	ND	ND	ND	ND	ND	ND	ND

Table S2. Plasma concentrations of TMP (ng/mL) at various time points after a single oral dose of TMP NPs/SMZ in rats (n=6).

time（h）	1	2	3	4	5	6	Mean	SD
0.083	219.20	274.14	188.68	250.35	193.97	217.93	224.05	32.92
0.25	436.26	456.73	317.08	472.89	356.43	333.47	395.48	67.71
0.5	638.73	534.18	439.75	588.58	488.69	478.94	528.15	74.44
1	472.19	689.54	587.97	476.37	549.35	573.96	558.23	80.69
2	383.27	548.45	453.06	393.93	383.34	405.68	427.96	64.48
4	217.92	402.06	310	316.1	272.12	313.60	305.30	60.51
6	105.46	307.36	241.52	236.37	205.86	216.42	218.83	65.86
8	44.45	178.64	110.86	123.59	169.47	147.54	129.09	48.89
12	9.28	68.17	58.27	70.8	77.4	69.33	58.88	25.06
24	ND	ND	ND	ND	ND	ND	ND	ND
36	ND	ND	ND	ND	ND	ND	ND	ND
48	ND	ND	ND	ND	ND	ND	ND	ND
72	ND	ND	ND	ND	ND	ND	ND	ND

Table S3. Plasma concentrations of SMZ (ng/mL) at various time points after a single oral dose of TMP/SMZ in rats (n=6).

time（h）	1	2	3	4	5	6	Mean	SD
0.083	892.66	657.3	795.22	933.58	598.35	385.26	710.40	205.58
0.25	1538.42	1134.43	1389.67	1683.23	1016.39	965.22	1287.89	293.53
0.5	2751.63	2381.49	2698.72	2863.6	2104.53	1466.1	2377.68	526.24
1	3218.04	2960.54	3167.78	3284.51	2690.86	1876.55	2866.38	530.81
2	4779.31	4178.23	4325.06	4360.46	3968.42	3392.48	4167.33	464.06
4	6431.52	5823.59	5432.19	6157.8	5258.58	4590.87	5615.76	665.45
6	5846.49	6157.35	7169.27	5289.67	4548.27	5669.83	5780.15	877.11
8	5237.76	5213.78	6341.64	4632.17	3651.62	4785.17	4977.02	883.02
12	3013.98	2932.65	4297.58	2867.55	1926.81	2752.5	2965.18	763.11
24	1738.67	1489.61	2319.57	1365.24	896.40	1406.38	1535.98	471.65
36	953.25	733.42	1015.83	756.69	446.25	869.44	795.81	203.09
48	518.91	314.96	657.34	381.11	36.76	473.29	397.06	212.23
72	26.78	ND	68.77	ND	ND	22.5	39.35	25.57

Table S4. Plasma concentrations of SMZ (ng/mL) at various time points after a single oral dose of TMP NPs/SMZ in rats (n=6).

time（h）	1	2	3	4	5	6	Mean	SD
0.083	1237.56	989.5	1533.49	638.45	1300	1943.45	1273.74	447.74
0.25	2198	1620.45	2613.26	1432.88	2089.2	3030.25	2164.01	598.16
0.5	2716.69	2193.44	3106.54	1955.05	2881.24	3689.67	2757.11	627.67
1	3336.45	2761.12	3838.67	2415.3	3135.26	4543.25	3338.34	765.32
2	4207.86	3537.68	5179.4	3174.46	4333.24	5858.58	4381.87	1001.98
4	5499.67	4258.42	6378.68	3923.83	5258.4	6978.24	5382.87	1179.76
6	6157.88	5698.47	5469.12	5219.57	6568.21	5678.9	5798.69	487.76
8	4932.67	4378.76	4788.43	3889.67	5347.14	4875.22	4701.98	504.23
12	2925.78	2517.4	2658.65	2351.23	3478.39	2917.26	2808.12	397.65
24	1680.55	1154.47	1567.88	1119.4	2018.77	1752.63	1548.95	352.14
36	1013.5	768	984.25	658.82	1652.28	1156.66	1038.92	349.74
48	613.83	168.58	637.41	105.47	926.53	688.45	523.38	319.84
72	40.22	ND	38.50	ND	167.44	54.37	75.13	61.95

Table S5. Tissue distribution concentrations of TMP (ng/g) at various time points after single oral dose of two TMP/SMZ formulations in rats (x±s, n=6).

A: TMP/SMZ group, B: TMP NPs/SMZ group.

Time Tissue		Heart	Liver	Spleen	Lung	Kidney	Stomach	Brain	Small Intestine
0.5 h	A	219±20	379±63	485±83	646±72	1187±78	1399±153	35±8	1153±109
	B	269±54	1231±119*	765±57*	1484±74*	1245±72	1905±107*	86±10*	1931±111*
1h	A	121±41	275±67	289±35	426±28	782±111	466±85	20±4	687±69
	B	86±7	741±93*	337±87	839±52*	1448±71*	1645±106*	23±5	958±92*
2h	A	12±7	84±13	64±10	92±8	257±55	416±39	ND	163±11
	B	28±9*	348±55*	128±22*	477±61*	500±80*	928±61*	4±1	904±86*
6h	A	5±3	ND	ND	ND	161±18	312±39	ND	ND
	B	14±4*	148±25*	46±9*	73±12*	236±55*	822±36*	ND	376±84*
12h	A	ND	ND	ND	ND	60±11	136±29	ND	ND
	B	ND	15±5*	13±4*	ND	138±46*	616±53*	ND	26±9*

Note:*Statistical significance compared with A group is $p < 0.05$.

Table S6. Tissue distribution concentrations of SMZ(ng/g) at various time points after single oral dose of two TMP/SMZ formulations in rats (x±s, n=6).

A: TMP/SMZ group, B: TMP NPs/SMZ group.

Time Tissue		Heart	Liver	Spleen	Lung	Kidney	Stomach	Brain	Small Intestine
0.5 h	A	1571±111	824±117	667±74	1601±116	2365±254	4688±699	431±50	1772±119
	B	1514±140	1386±190*	1190±94*	2241±171*	2432±235	4502±449	785±97*	1951±103*
1h	A	1845±92	1343±185	1125±115	1271±183	2657±155	3194±253	1208±102	1108±120
	B	1139±91*	1234±173	1024±48	1216±119	1941±116*	3317±324	1416±238*	1133±133
2h	A	1389±104	373±100	1779±71	1202±131	2651±190	2719±300	1300±111	565±55
	B	940±90*	833±78*	1711±50	1192±86	2224±471*	2844±500	1427±92	974±38*
6h	A	866±78	197±73	702±49	698±29	1590±121	1699±223	547±67	475±78
	B	762±68*	315±47*	532±58*	855±65*	1575±141	1319±87*	628±64	661±92*
12h	A	570±61	65±5	229±33	520±66	893±103	1175±84	260±54	109±22
	B	216±48*	131±39*	193±4*	219±36*	543±99*	1055±76*	305±59	206±21*

Note:*Statistical significance compared with A group is $p < 0.05$.

Table S7. Urinary and fecal excretion rates of TMP in rats following single-dose oral administration of two TMP/SMZ formulations (n=6).

Matrix	Time Interval (h）	Cumulative Excretion Rate ($\bar{x}$±s,%)	
		TMP/SMZ	TMP NPs/SMZ
Urine	0-4	14.88±7.94	36.99±8.08
	4-8	13.94±11.23	14.33±4.00
	8-12	7.71±1.60	9.74±1.61
	12-24	8.22±1.72	9.34±4.51
	24-36	3.59±1.63	2.45±0.75
	36-48	1.47±0.52	0.82±0.35
	48-72	0.35±0.14	0.22±0.12
Feces	0-4	0.18±0.06	0.35±0.13
	4-8	2.43±0.49	6.07±0.93
	8-12	2.92±0.36	6.14±1.37
	12-24	ND	0.41±0.11
	24-36	ND	ND

Table S8. Urinary and fecal excretion rates of SMZ in rats following single-dose oral administration of two TMP/SMZ formulations (n=6).

Matrix	Time Interval (h）	Cumulative Excretion Rate ($\bar{x}$±s,%)	
		TMP/SMZ	TMP NPs/SMZ
Urine	0-4	20.33±10.79	29.19±6.53
	4-8	21.14±11.69	21.61±5.62
	8-12	10.35±2.36	6.22±2.09
	12-24	14.64±2.47	9.35±3.25
	24-36	4.20±1.36	3.65±0.70
	36-48	1.43±0.55	2.26±0.52
	48-72	0.24±0.07	0.31±0.11
Feces	0-4	1.90±0.35	1.94±0.39
	4-8	1.19±0.20	4.26±0.59
	8-12	1.48±0.31	3.35±0.52
	12-24	ND	0.70±0.11
	24-36	ND	ND

Table S9. Liquid chromatography gradient elution profile.

Time (minutes)	Mobile phase A (%)	Mobile phase B (%)
0	80	20
1	80	20
7	20	80
8	20	80
9	80	20
13	80	20
